# Genomic and secretomic insight into lignocellulolytic system of an endophytic bacterium *Pantoea ananatis* Sd-1

**DOI:** 10.1186/s13068-016-0439-8

**Published:** 2016-02-02

**Authors:** Jiangshan Ma, Keke Zhang, Hongdong Liao, Stanton B. Hector, Xiaowei Shi, Jianglin Li, Bin Liu, Ting Xu, Chunyi Tong, Xuanming Liu, Yonghua Zhu

**Affiliations:** Hunan Province Key Laboratory of Plant Functional Genomics and Developmental Regulation, College of Biology, Hunan University, Changsha, 410008 Hunan People’s Republic of China; Department of Genetics, Institute for Plant Biotechnology, Stellenbosch University, Private Bag X1, Matieland, Stellenbosch, 7602 South Africa; State Key Laboratory of Chemo/Biosensing and Chemometrics, College of Biology, Hunan University, Changsha, 410008 Hunan People’s Republic of China; DNA Sequencing Unit, Central Analytical Facility, Stellenbosch University, Private Bag X1, Matieland, Stellenbosch, 7602 South Africa

**Keywords:** Endophytic bacterium, *Pantoea ananatis* Sd-1, Lignocellulose degradation, CAZy, Quantitative real-time PCR, Secretome, Enzymes activities

## Abstract

**Background:**

Exploring microorganisms especially bacteria associated with the degradation of lignocellulosic biomass shows great potentials in biofuels production. The rice endophytic bacterium *Pantoea ananatis* Sd-1 with strong lignocellulose degradation capacity has been reported in our previous study. However, a comprehensive analysis of its corresponding degradative system has not yet been conducted. The aim of this work is to identify and characterize the lignocellulolytic enzymes of the bacterium to understand its mechanism of lignocellulose degradation and facilitate its application in sustainable energy production.

**Results:**

The genomic analysis revealed that there are 154 genes encoding putative carbohydrate-active enzymes (CAZy) in *P. ananatis* Sd-1. This number is higher than that of compared cellulolytic and ligninolytic bacteria as well as other eight *P. ananatis* strains. The CAZy in *P. ananatis* Sd-1 contains a complete repertoire of enzymes required for cellulose and hemicellulose degradation. In addition, *P. ananatis* Sd-1 also possesses plenty of genes encoding potential ligninolytic relevant enzymes, such as multicopper oxidase, catalase/hydroperoxidase, glutathione S-transferase, and quinone oxidoreductase. Quantitative real-time PCR analysis of parts of genes encoding lignocellulolytic enzymes revealed that they were significantly up-regulated (at least *P* < 0.05) in presence of rice straw. Further identification of secretome of *P. ananatis* Sd-1 by nano liquid chromatography–tandem mass spectrometry confirmed that considerable amounts of proteins involved in lignocellulose degradation were only detected in rice straw cultures. Rice straw saccharification levels by the secretome of *P. ananatis* Sd-1 reached 129.11 ± 2.7 mg/gds. Correspondingly, the assay of several lignocellulolytic enzymes including endoglucanase, exoglucanase, β-glucosidase, xylanase-like, lignin peroxidase-like, and laccase-like activities showed that these enzymes were more active in rice straw relative to glucose substrates. The high enzymes activities were not attributed to bacterial cell densities but to the difference of secreted protein contents.

**Conclusion:**

Our results indicate that *P. ananatis* Sd-1 can produce considerable lignocellulolytic enzymes including cellulases, hemicellulases, and ligninolytic relevant enzymes. The high activities of those enzymes could be efficiently induced by lignocellulosic biomass. This identified degradative system is valuable for the lignocellulosic bioenergy industry.

**Electronic supplementary material:**

The online version of this article (doi:10.1186/s13068-016-0439-8) contains supplementary material, which is available to authorized users.

## Background

Lignocellulose in vascular plant cell walls is the largest reservoir of organic polymers in terrestrial ecosystems and is a potential biofuel feedstock alternative to petroleum [1]. The main components of lignocellulose are polysaccharides and lignin. They are bound together in a matrix and the recalcitrance of structure gives rise to a major bottleneck in the efficient conversion of lignocellulosic biomass to biofuels [2]. Thus, exploring highly efficient lignocellulose decomposition microorganisms has attracted significant research attention [3]. During the past decade, only a limited number of microorganisms, such as various types of fungi, were found to breakdown the complex lignocellulose polymer structures [4]. Recently, bacterial systems have attracted an increasing amount of attention due to their unique mechanisms for lignocellulose decomposition and the high specificity of their lignocellulolases. Bacteria also have great advantages over fungi due to their rapid growth, wide range of environmental adaptability, and facilitative genetic manipulations [5, 6].

Bacterial candidates for degrading lignocellulosic polysaccharides have been identified by genomic and proteomic analysis. *Caldicellulosiruptor bescii* DSM 6725, *Clostridium thermocellum* ATCC 27405 and *Streptomyces* SDPB6 had been sequenced, and a lot of genes in these bacteria strains encoding putative carbohydrate-active enzymes (CAZy) were identified, and their presence were partially confirmed by proteomic analysis results [7–9]. Recently, qualitative and quantitative proteomic analysis methods were used to demonstrate that lignocellulolytic enzymes in *Thermobifida fusca* were highly up-regulated in the presence lignocellulosic biomass [10–12]. However, bacterium that can degrade both lignocellulosic polysaccharides and lignin was rarely found [13, 14], let alone a comprehensive research of the lignocellulolytic system, including cellulases, hemicellulases, and ligninolytic enzymes in the bacteria was reported.

Endophytes which spend the whole or part of its life cycle colonizing inside the healthy host plant tissues have been the subject of research attention worldwide, due to its wide distribution and the ability to produce a wide variety of secondary metabolites. They can secrete a large number of proteins, including lignocellulolytic enzymes, to assist their adaption and survival within higher plants [15]. A few endophytic fungi demonstrating lignocellulosic biomass degrading ability have already been discovered [16, 17]. However, endophytic bacteria as potential lignocellulose degradation resources have been largely overlooked.

We have isolated a rice endophytic bacterium *Pantoea* sp. Sd-1 (China General Microbiological Culture Collection Centre, no. 6698). It showed strong lignocellulosic biomass degradation capacity with 54.5 % of rice straw weight lost (cellulose, hemicellulose, and lignin content reduced 80.1, 59.6, and 33.1 %, respectively) after 6 days treatment and kraft lignin content reduced 69.1 % after 4 days incubation [18]. It was further identified as *Pantoea ananatis* Sd-1 by whole genome sequencing analysis. Generally the *Pantoea* genus is recognized for its plant pathogenesis [19]. However, it has also been reported as lignocellulose degradation bacterium. *Pantoea* sp. SL1_M5 isolated from invasive woodwasp *Sirex noctilio* and displayed degrading ability toward cellulose and its derivatives [20]. A few other *P. ananatis* strains were also reported to possess carbohydrate degradation enzymes [14, 21]. *Pantoea* sp. RCT2 isolated from pulp paper mill effluent and showed ligninolytic enzyme activity and strong pulp paper mill effluent decolorisation capacity [22]. The *Pantoea* sp NII-153 isolated from soil showed high strength lignin derivative degradation ability [23]. These indicated that *Pantoea* genus strains might be potential resources for lignocellulosic biomass biotransformation industry.

*Pantoea ananatis* Sd-1 possesses the degradation ability for both lignocellulosic polysaccharides and lignin. Uncovering corresponding genes and enzymes in its lignocellulolytic system will be essential for its efficient application in biofuel industry. In the present study, we sequenced the whole genome of *P. ananatis* Sd-1 and compared its characteristics to those of five typical cellulolytic and ligninolytic bacteria as well as eight other *P. ananatis* strains. We identified candidate genes involved in lignocellulose degradation through genomic analysis, and some of them were confirmed by analyzing their expression levels. Comparative proteome analysis was also conducted to differentiate the enzyme cocktails produced by *P. ananatis* Sd-1 when it grew on different carbon sources. In addition, this secretome was also evaluated for rice straw saccharification. Furthermore, several typical lignocellulolytic enzymes activity were detected and compared.

## Results

### Genomic analysis of *P. ananatis* Sd-1

The assembled genome of *P. ananatis* Sd-1 [GenBank: AZTE00000000] comprises a total of 4927,500 bp containing 4332 protein coding sequences (CDS) (detailed results were shown in Additional file 1: Table S1). To identify genes involved in lignocellulose degradation, CDS were analyzed using dbCAN carbohydrate-active enzymes (CAZy) annotation algorithm. Results indicated that 154 genes have multiple domains assigned to CAZy families, including 59 glycoside hydrolases (GHs), 25 carbohydrate esterases (CEs), 2 polysaccharide lyases (PLs), 9 enzymes with auxillary activities (AAs), and 11 carbohydrate binding modules (CBMs); 28 of these proteins contain signal peptides and are predicted to be extracellular proteins (Additional file 2: Table S2).

The genome characteristics of *P. ananatis* Sd-1 was compared with five well-studied cellulolytic and ligninolytic bacteria: *Pantoea* sp. SL1_M5 [GenBank: ADWZ00000000] [20], *C. bescii* DSM 6725 [GenBank: CP001393] [7], *C. thermocellum* ATCC 27405 [GenBank: CP000568] [8], *Sphingobium* sp. SYK-6 [GenBank: AP012222] [24], and *Enterobacter lignolyticus* SCF1 [GenBank: CP002272] [25], and eight reported *P. ananatis* strains with potential carbohydrate degradation ability [21]: *P. ananatis* AJ13355 [GenBank: AP012032], *P. ananatis* LMG 20103 [GenBank: CP001875], *P. ananatis* LMG 5342 [GenBank: HE617160], *P. ananatis* PA13 [GenBank: CP003085], *P. ananatis* B1-9 [GenBank: CAEJ00000000], *P. ananatis* LMG 2665 [GenBank: JMJJ00000000], *P. ananatis* PA4 [GenBank: JMJK00000000], and *P. ananatis* BD442 [GenBank: JMJL00000000]. The number of putative CAZy genes in *P. ananatis* Sd-1 (154) is the highest, and the percentage of putative CAZy genes (3.6 %) is the second highest among all compared strains (Table 1). *P. ananatis* Sd-1, the eight other *P. ananatis* strains and the cellulolytic bacterium *C. bescii* DSM 6725 possess similar numbers of GHs and CBMs, which are only less than those of the cellulolytic bacterium *C. thermocellum* ATCC 27405. The amounts of CEs and AAs in *P. ananatis* Sd-1 are significantly higher than those in all compared strains, including the eight *P. ananatis* strains. It is also worth mentioning that AAs were absent from the five other cellulolytic and ligninolytic bacteria as well as two *P. ananatis* strains. We only found one AA in *P. ananatis* AJ13355, *P. ananatis* LMG 20103, *P. ananatis* B1-9, *P. ananatis* LMG 2665, *P. ananatis* PA4, and *P. ananatis* BD442.

**Table 1:** Comparison of genome of *P. ananatis* Sd-1 with cellulolytic, ligninolytic bacteria, and other *P. ananatis* strains

Species	Genome size (bp)	Protein coding genes	Total CAZy^b^	% CAZy^a^	GHs^c^	CEs^d^	CBMs^e^	PLs^f^	AAs^g^
*P. ananatis* Sd-1	4,927,500	4332	154	3.6	59	25	11	2	9
*P. ananatis* AJ13355	4,877,280	4222	108	2.6	52	4	9	0	1
*P. ananatis* LMG 2013	4,703,370	4076	105	2.6	51	4	8	0	1
*P. ananatis* LMG 5342	4,908,140	4345	108	2.5	55	4	10	0	0
*P. ananatis* PA13	4,867,130	4403	112	2.5	59	4	10	0	0
*P. ananatis* B1-9	5,105,560	4672	129	2.8	58	9	10	2	1
*P. ananatis* LMG 2665	4,633,915	4397	123	2.8	55	9	10	2	1
*P. ananatis* PA4	5,163,480	4662	131	2.8	60	7	13	2	1
*P. ananatis* BD442	4,798,550	4292	125	2.9	55	8	11	2	1
*Pantoea* sp. SL1_M5	4,924,830	4626	41	0.9	28	2	3	0	0
*C. thermocellum* ATCC 27405	3,843,300	3263	145	4.4	74	16	90	4	0
*C. bescii* DSM 6725	2,931,660	2662	88	3.3	49	6	18	3	0
*Sphingobium* sp. SYK-6	4,348,130	3939	70	1.7	21	1	3	0	0
*E. lignolyticus* SCF1	4,814,050	4350	94	2.1	42	5	7	0	0

^a^Percentage of CAZy genes in protein coding genes

^b^ *CAZy* carbohydrate-active enzymes

^c^ *GHs* glycoside hydrolases

^d^ *CEs* carbohydrate esterases

^e^ *CBMs* carbohydrate bingding modules

^f^ *PLs* polysaccharide lyases

^g^ *AAs* auxillary activities

In *P. ananatis* Sd-1, there are 10 cellulase genes including: one endoglucanase (GH8) gene, one exoglucanase (GH10) gene, and eight β-glucosidase genes (GH1, 3) (Table 2). Among those cellulases, four proteins sequences including endoglucanase and exoglucanase exhibit potential secretion signals. The number of cellulase genes in *P. ananatis* Sd-1 is the same as that found in the eight compared *P. ananatis* strains, which is only less than that possessed by the cellulolytic bacterium *C. thermocellum* ATCC 27405 (16).

**Table 2:** Predicted CAZymes and other potential lignocellulolytic enzymes in the *P. ananatis* Sd-1 genome

Enzymes	Specific activity	Locus tag	CAZy modules	Signal peptides
Cellulose and hemicellulose degradation relevant enzymes	Endoglucanases	Y903_RS0123720	GH8	Y
	Exoglucanases	Y903_RS0108720	GH10	Y
	β-glucosidases	Y903_RS0109910	GH1	N
		Y903_RS0123340	GH1	Y
		Y903_RS0115000	GH1	N
		Y903_RS0103625	GH1	N
		Y903_RS0104945	GH1	N
		Y903_RS0106975	GH1	N
		Y903_RS0113180	GH3	Y
		Y903_RS0116520	GH3	N
	Oxidoreductase	Y903_RS0100765	GH109	N
		Y903_RS0119930	GH109	N
		Y903_RS0103245	GH109	N
		Y903_RS0108420	GH109	N
	α-N-arabinofuranosidase	Y903_RS0119145	GH43	N
		Y903_RS0120675	GH51	N
	α-mannosidase	Y903_RS0105780	GH38	N
	Galactosidase	Y903_RS0108500	GH2	N
		Y903_RS0110160	GH4	N
	Esterase	Y903_RS0100380	CE1	N
		Y903_RS0104455	CE10	Y
		Y903_RS0104990	CE16	N
		Y903_RS0120815	CE10	Y
		Y903_RS0117780	CE10	Y
	Acyl-CoA esterase	Y903_RS0113655	CE1	N
	Acyl-CoA thioesterase	Y903_RS0114420	CE3	N
	Carboxylesterase	Y903_RS0117855	CE1	N
		Y903_RS0116340	CE10	N
Lignin degradation relevant enzymes	Multicopper oxidase	Y903_RS0111680	–	Y
		Y903_RS0119240	–	Y
	Catalase/hydroperoxidase	Y903_RS0115335	AA2	N
	GMC family oxidoreductase	Y903_RS0107765	AA3	N
	Quinone oxidoreductase	Y903_RS0112360	–	N
		Y903_RS0117910	–	N
		Y903_RS0118595	–	N
		Y903_RS0110255	–	N
		Y903_RS0120835	–	N
		Y903_RS0120850	–	N
		Y903_RS0120855	–	N
		Y903_RS0120880	–	N
		Y903_RS0105425	–	N
		Y903_RS0107980	–	N
	Glutathione S-transferase	Y903_RS0123560	–	N
		Y903_RS0108490	–	N
		Y903_RS0107875	–	N
		Y903_RS0120970	–	N

Similar to the eight compared *P. ananatis* strains, there are five GHs containing CBM domains in *P. ananatis* Sd-1 (Additional file 3: Figure S1), which are surprisingly not related to cellulose degradation. However, there are three cellulase genes containing multiple CBM domains in cellulolytic bacterium *C. bescii* DSM 6725, and the majority of cellulase system-related genes comprise multiple CBM domains in cellulolytic bacterium *C. thermocellum* ATCC 27405.

Although there are no typical xylanase genes in *P. ananatis* Sd-1, we found nine esterases (CE1, 3, 10, 16) genes in it (Table 2). Among these esterases, Y903_RS0120815, Y903_RS0117780, and Y903_RS0104455 which all belong to the CE10 family possess signal peptide sequences. In addition, the number of esterases genes in *P. ananatis* Sd-1 is higher than that of all of the compared strains. Furthermore, some GHs involved in hemicellulosic polysaccharides hydrolysis, such as α-N-arabinofuranosidase (GH43, 51), α-mannosidase (GH38), and galactosidase (GH2, 4) have also been found in *P. ananatis* Sd-1 (Table 2).

**Fig. 1:**
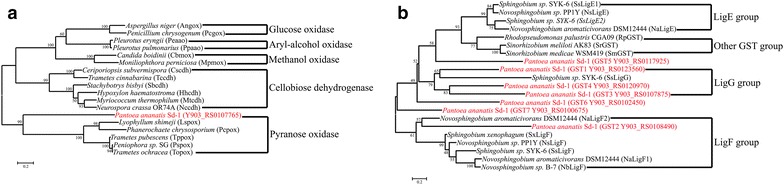
Phylogenetic tree analysis of GMC family oxidoreductase (**a**) and glutathione S-transferases (**b**) amino acid sequences. The tree was generated by the maximum likelihood algorithm (1000 bootstrap trials) using MEGA 5.1. The *scale bar* represents the number of amino acid substitutions per site. The *red color* indicates amino acid sequences selected from *P. ananatis* Sd-1. *Gox* glucose oxidase, *aao* aryl-alcohol oxidase, *mox* methanol oxidase, *cdh* cellobiose dehydrogenase, *pox* pyranose oxidase, *GST* glutathione S-transferase. Angox [GeneBank: CAC12802]; Pcgox [GeneBank: AFA42947]; Peaao [GeneBank: AAC72747]; Ppaao [GeneBank: AAF31169]; Cbmox [GeneBank: AAA34321]; Mpmox [GeneBank: AFO55203]; Cscdh [GeneBank: ACF60617]; Tccdh [GeneBank: ADX41688]; Sbcdh [GeneBank: ADT70778]; Hhcdh [GeneBank: ADT70776]; Mtcdh [GeneBank: ABS45567]; Nccdh [GeneBank: EAA27355]; Lspox [GeneBank: BAD12079]; Pcpox [GeneBank: AAS93628]; Tppox [GeneBank: AAW57304]; Pspox [GeneBank: AAO13382]; Topox [AAP40332]; SsLigE [GeneBank: BAK65541]; NsLigE [GeneBank: CCA92088]; SsLigP [GeneBank: BAK67935]; NaLigE [GeneBank: ABD26841]; RpGST [GeneBank: CAE29781]; SrGST [GeneBank: AEG52712]; SmGST [GeneBank: YP_001326465]; SsLigF1 [GeneBank: BAK65540]; NsLigF1 [GeneBank: CCA92087]; SxLigF2 [GeneBank: WP_019052363]; NaLigF1 [GeneBank: ABD26530]; NbLigF1 [GeneBank: WP_022675760]; NaLigF2 [GeneBank: ABD27301]; SsLigG [GeneBank: BAK65542]

Several genes encoding proteins potentially involved in lignin degradation were identified in *P. ananatis* Sd-1 (Table 2). These include multicopper oxidases, catalase/hydroperoxidase (AA2), glucose–methanol–choline (GMC) family oxidoreductase (AA3), glutathione S-transferases (GSTs), and quinone oxidoreductases. Phylogenetic analysis revealed that the GMC family oxidoreductase was closely related to pyranose oxidases (Fig. 1a). There are seven GST in *P. ananatis* Sd-1. Phylogenetic analysis indicated that *gst*1 (Y903_RS0123560), *gst*3 (Y903_RS0107875), and GST4 (Y903_RS0120970) were closely related to LigG, and GTS2 (Y903_RS0108490) belongs to the LigF group (Fig. 1b). Our analyses indicate that these four GSTs might possess β-etherase activity. Among the above-mentioned enzymes potentially involved in lignin degradation, only the multicopper oxidases are predicted to be extracellular proteins.

### Gene expression analysis for *P. ananatis* Sd-1 cultured in rice straw or glucose medium

**Fig. 2:**
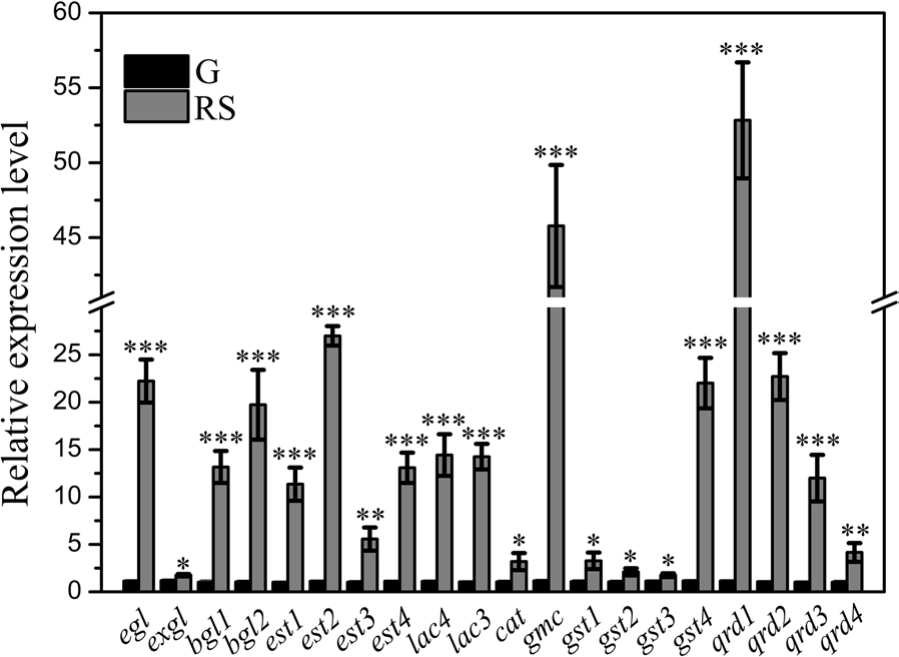
Lignocellulolytic enzyme genes relative expression levels of *P. ananatis* Sd-1 in the presence of different substrates. *G* glucose substrate, *RS* rice straw substrate. *egl* (Y903_RS0123720): endoglucanase, *exg*l (Y903_RS0108720): exoglucanase, *bgl*1 (Y903_RS0116520): β-glucosidase 1, *bgl*2 (Y903_RS0113180): β-glucosidase 2, *est*1 (Y903_RS0120815): esterase 1, *est*2 (Y903_RS0117780): esterase 2, *est*3 (Y903_RS0100380): esterase 3, *est*4 (Y903_RS0113655): esterase 4, *lac*4 (Y903_RS0119240): multicopper oxidase 1, *lac*3 (Y903_RS0111680): multicopper oxidase 2, *cat* (Y903_RS0115335): catalase/hydroperoxidase, *gmc* (Y903_RS0107765): GMC family oxidoreductase, *gst*1 (Y903_RS0123560): glutathione S-transferase 1, *gst*2 (Y903_RS0108490): glutathione S-transferase 2, *gst*3 (Y903_RS0107875): glutathione S-transferase 3, *gst*4 (Y903_RS0120970): glutathione S-transferase 4, *qrd*1 (Y903_RS0107980): quinone oxidoreductase 1, *qrd*2 (Y903_RS0118595): quinone oxidoreductase 2, *qrd*3 (Y903_RS0110255): quinone oxidoreductase 3, *qrd*4 (Y903_RS0112360): quinone oxidoreductase 4. The values represent the means of the three replicates with the standard deviation (SD). *Asterisks* represent significant differences from the glucose-containing medium (Statistical significance: * *P* < 0.05, ** *P* < 0.01, *** *P* < 0.001)

Expression profiles of selected *P. ananatis* Sd-1 lignocellulolytic enzymes genes were monitored in cultures grown on rice straw compared to cultures utilizing glucose as sole carbon source. Quantitative real-time PCR (qRT-PCR) investigation results of genes encoding endoglucanase (*egl* Y903_RS0123720), exoglucanase (*exg*l Y903_RS0108720), β-glucosidase (*bgl*1 Y903_RS0116520 and *bgl*2 Y903_RS0113180), esterase (*est*1 Y903_RS0120815, *est*2 Y903_RS0117780, *est*3 Y903_RS0100380 and *est*4 Y903_RS0113655), multicopper oxidase (*lac*3 Y903_RS0111680 and *lac*4 Y903_RS0119240), catalase/hydroperoxidase (*cat* Y903_RS0115335), GMC family oxidoreductase (*gmc* Y903_RS0107765), GST (*gst*1 Y903_RS0123560, *gst*2 Y903_RS0108490, *gst*3 Y903_RS0107875, and *gst*4 Y903_RS0120970), and quinone oxidoreductase (*qrd*1 Y903_RS0107980, *qrd*2 Y903_RS0118595, *qrd*3 Y903_RS0110255, and *qrd*4 Y903_RS0112360) are shown in Fig. 2. Transcript levels of all the genes of interest were significantly up-regulated (at least *P* < 0.05) in rice straw cultures compared to basic glucose cultures. Remarkably, transcript levels of several cellulase genes (*egl*, *bgl*1, and *bgl*2), hemicellulase genes (*est*1, *est*2, and *est*4), and ligninolytic-related genes (*lac*3, *lac*4, *gmc*, *gst*4, *qrd*1, *qrd*2, and *qrd*3) were increased more than tenfold in rice straw cultures.

### Identification of secretomes of *P. ananatis* Sd-1 grown on medium containing with or without rice straw

**Fig. 3:**
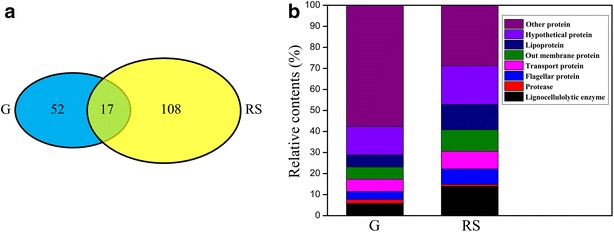
Secretome identification of *P. ananatis* Sd-1 in the presence of different substrates. **a** Venn diagram of the proteins identified in the *P. ananatis* Sd-1 secretome between media using rice straw (RS) and glucose substrates by nanoLC–MS/MS (G). **b** The relative contents of the various proteins identified by nanoLC–MS/MS in glucose-containing medium (G) and rice straw-containing medium (RS)

One-dimensional polyacrylamide gel electrophoresis (1D-PAGE) followed by nano liquid chromatography–tandem mass spectrometry (nanoLC–MS/MS) analysis was used to further identify the lignocellulolytic enzymes system in *P. ananatis* Sd-1 cultured in rice straw and glucose media, respectively (1D-PAGE gel picture was shown in Additional file 4: Figure S2). The results showed that the composition of the secreted proteins from *P. ananatis* Sd-1 differed significantly between the two media. The molecular weights of identified proteins along with their isoelectric points (pI) were shown in Additional file 5: Figure S3. Most of pI were in the range of 5.0–10.0. In total, 108 proteins were identified in rice straw culture and only 52 proteins were identified in glucose culture (detailed results were shown in Additional file 6). Among these proteins, 17 were found to be common in both cultures (shown in Fig. 3a). The presence of a few intracellular proteins was also detected, which could be a result of cell death and/or cell lysis during incubation during secretome extraction. Similar results were also reported in previous studies [12, 26].

Identified proteins were divided into the following categories: lignocellulolytic enzyme, protease, flagellar protein, transport protein, out membrane protein, lipoprotein, hypothetical protein, and other proteins. 15 of the 108 proteins (13.9 %) in the rice straw growth proteome may be putatively associated with lignocellulose degradation, while there might be only 3 (5.8 %) associated with glucose grown cells (Fig. 3b). Enzymes involved in polysaccharides degradation included cellulolytic and hemicellulolytic enzymes as well as other GHs were prevalent in rice straw cultures, such as one endoglucanase (GH8) (*egl* Y903_RS0123720), three β-glucosidases (GH1 and GH3) (*bgl*1 Y903_RS0123340, *bgl*2 Y903_RS0113180, and *bgl*3 Y903_RS0103625), one exoglucanase (GH10) (*exg*l Y903_RS0108720) two esterases (CE10) (*est*1 Y903_RS0120815 and *est*2 Y903_RS0104455), one β-N-acetylhexosaminidase (GH3) (Y903_RS0108355), and one glycosyl hydrolase (GH33) (Y903_RS0104515). In conjunction with the incidence of carbohydrate degradative enzymes in rice straw cultures, lignin degradation relevant proteins were only represented in rice straw cultures including one catalase/hydroperoxidase (AA2) (*cat* Y903_RS0115335) and one multicopper oxidase (*lac*4 Y903_RS0119240) (Table 3). Among these enzymes, most possessed signal peptides, except for one β-glucosidase (Y903_RS0103625), one glycosyl hydrolase (Y903_RS0104515), and one catalase/hydroperoxidase (Y903_RS0115335). There were only three CAZymes identified in glucose cultures: one hypothetical protein (CE1) (Y903_RS0116730), one translocation protein TolB (PL22) (Y903_RS0113490), and one lysozyme (GH24) (Y903_RS0115180).

**Table 3:** Identification of main lignocellulolytic proteins in the secretome of *P. ananatis* Sd-1 in rice straw culture

Locus tag	Identified protein description	Score	Unique peptides	Peptides	Protein mass (kDa)	Isoelectric point	Signal peptides
Y903_RS0108720	exoglucanase	12.84	5	5	41.6	8.72	Y
Y903_RS0103625	beta-glucosidase	12.06	3	3	44.2	5.79	N
Y903_RS0113180	beta-glucosidase	10.31	3	3	84	6.48	Y
Y903_RS0123340	beta-glucosidase	22.52	5	5	53.2	6.33	Y
Y903_RS0123720	endoglucanase	10.8	4	4	38.6	6.55	Y
Y903_RS0104515	glycosyl hydrolase	12.79	4	4	43.8	5.23	N
Y903_RS0104455	esterase	6.93	3	3	37.8	5.75	Y
Y903_RS0120815	esterase	7.74	2	2	33.1	9.60	Y
Y903_RS0108355	beta-N-acetylhexosaminidase	8.47	3	3	88.4	6.26	Y
Y903_RS0115335	catalase/hydroperoxidase	5.95	2	2	80	5.71	N
Y903_RS0119240	multicopper oxidase	11.23	4	4	58.1	6.25	Y

### Comparison of enzymes activities from *P. ananatis* Sd-1 grown on medium containing with or without rice straw

**Fig. 4:**
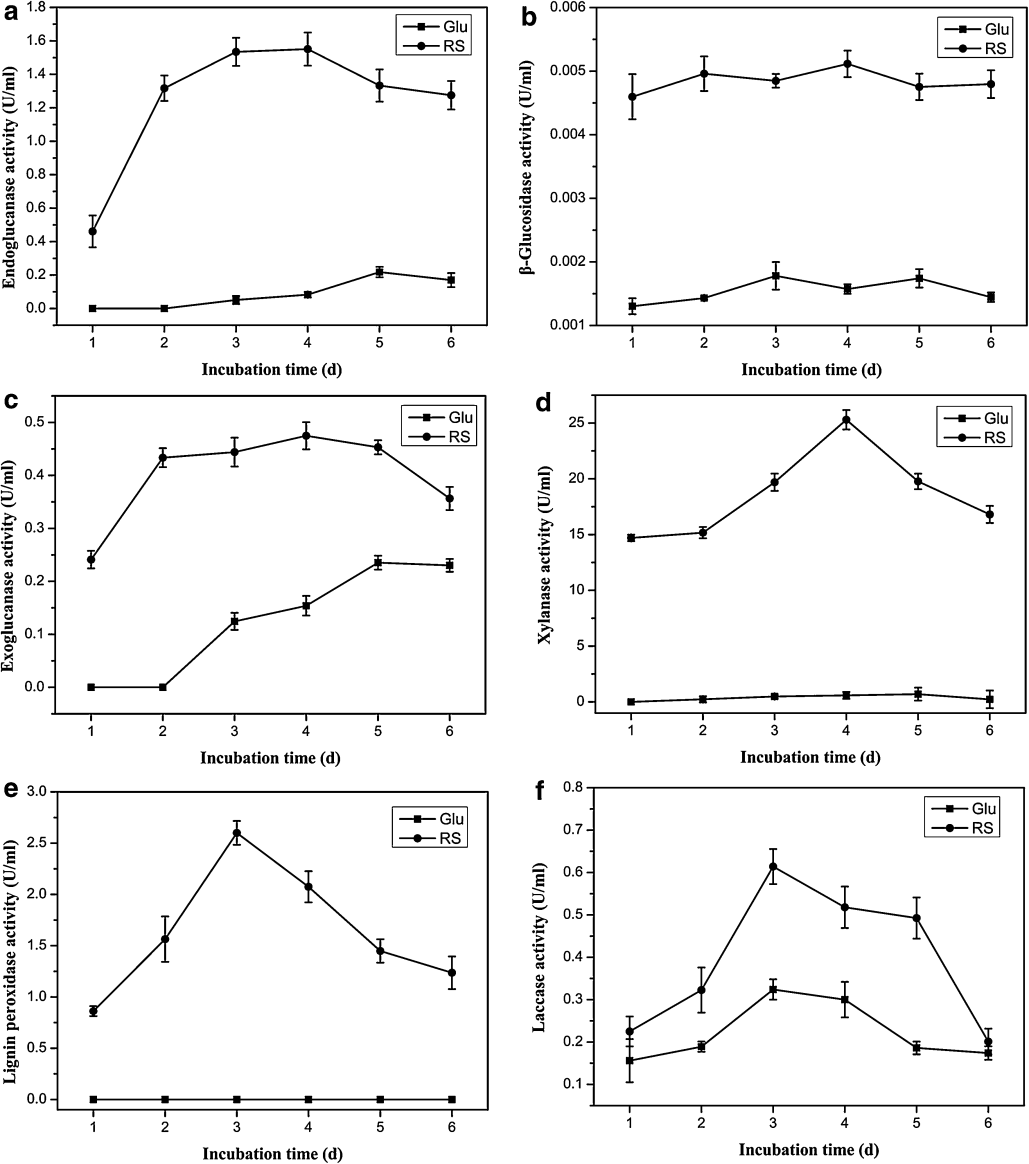
Lignocellulolytic enzyme activities of *P. ananatis* Sd-1 in the presence of different substrates. The activities of endoglucanase, β-glucosidase, exoglucanase, xylanase-like, lignin peroxidase-like, laccase-like are listed in **a**–**f**, respectively. *G* glucose substrate, *RS* rice straw substrate. Data are presented in triplicate from experiments as the mean standard deviation

We detected several lignocellulolytic enzymes activities displayed by *P. ananatis* Sd-1 to further confirm its degradative ability. As presented in Fig. 4, the lignin peroxidase-like activity was only detected in rice straw cultures. The activities of endoglucanase, β-glucosidase, xylanase-like, exoglucanase, and laccase-like for rice straw were significantly higher than those for glucose, especially for the first three enzymes. However, manganese peroxidase-like activity remained undetected in both cultures.

**Fig. 5:**
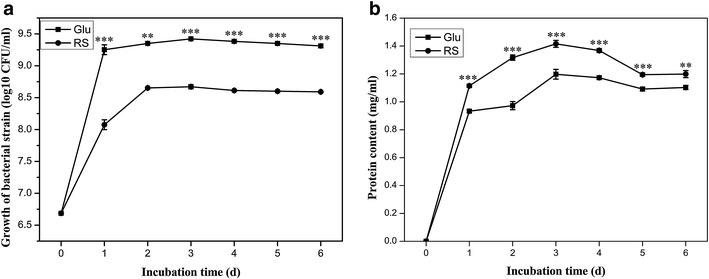
Growth versus protein content of *P. ananatis* Sd-1 in the presence of different substrates. **a** Growth of Sd-1 in the presence of glucose and rice straw substrates. **b** Protein content produced by Sd-1 in the presence of glucose and rice straw substrates. *G* glucose substrate, *RS* rice straw substrate. Data are presented in triplicate from experiments as the mean standard deviation. *Asterisks* represent significant differences from the glucose-containing medium (Statistical significance: * *P* < 0.05, ** *P* < 0.01, *** *P* < 0.001)

To investigate whether higher enzymes activities were caused by increases in bacterial growth or in the content of secreted proteins, *P. ananatis* Sd-1 grew on rice straw and glucose medium were assayed. In Fig. 5a, cell density of *P. ananatis* Sd-1 in glucose cultures was significantly higher compared to growth in rice straw cultures (at least *P* < 0.01). Cultures grown separately on glucose and rice straw reached saturation on day 3 with the value of 9.42 ± 0.01 log10 CFU/ml and 8.67 ± 0.03 log10 CFU/ml, respectively. In contrast, the amount of secreted protein contents in rice straw was significantly higher compared to that of glucose cultures (at least *P* < 0.01). The maximum protein contents of both cultures were reached at day 3 with the value of 1.19 ± 0.03 mg/ml and 1.42 ± 0.02 mg/ml, respectively (Fig. 5b).

### Hydrolysis of rice straw by secretome of *P. ananatis* Sd-1 from rice straw culture

**Fig. 6:**
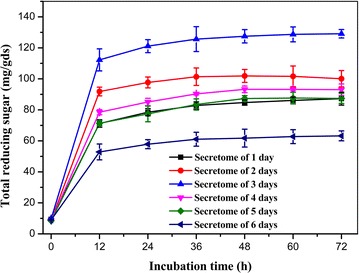
Reducing sugar released from rice straw by the enzymatic hydrolysis of *P. ananatis* Sd-1 secretome. Secretome of *P. ananatis* Sd-1 secretome was extracted from rice straw cultures at every 24-h interval

To further assess the lignocellulose degrading ability, the secretome of *P. ananatis* Sd-1 were extracted from rice straw cultures at different time intervals and evaluated for rice straw saccharification. As depicted in Fig. 6, with increased incubation, a basically increase in reducing sugar was released from rice straw. The highest yield of released reducing sugar was detected from the third day’s secretome with a value of 129.11 ± 2.7 mg/gds. And the sixth day’s secretome produced the least reducing sugar.

## Discussion

Lignocellulose degrading microorganisms and their degradative systems possess great potential for biofuels production [27]. To date few microorganisms with the exception of white-rot fungi have been reported to have the ability to degrade cellulose, hemicellulose, and lignin, simultaneously [28]. However, currently there is no commercial product for lignocellulose biodegradation, in part due to the practical challenges of fungal enzyme expression and fungal genetic manipulation [29]. Recent work indicates that the diverse bacterial lignocellulolytic systems may be more significant than previously thought, but their degradation catabolism has not been well characterized [30, 31]. Our previous study found that the endophytic bacterium *P. ananatis* Sd-1 had strong comprehensive ability to degrade cellulose, hemicellulose, and lignin [18]. Characterization of its degradation system will be very useful for utilization of plant lignocellulose for production of biofuels.

According to CAZy family classification, GHs, CEs, PLs, and CBMs reportedly play main roles in lignocellulosic polysaccharide degradation. Similarly, it has also been reported that AAs contribute largely to lignin breakdown [32]. Bioinformatic comparisons of CAZy in *P. ananatis* Sd-1 to other known cellulolytic and ligninolytic bacteria as well as *P. ananatis* strains confirmed its lignocellulolytic capabilities. Moreover, the biodegradation system in *P. ananatis* Sd-1 seems has its own characteristics.

In general, cellulose degradation is attributed to the synergistic action of three classes of glycosyl hydrolases: (1) endoglucanases, (2) exoglucanases, and (3) β-glucosidases [33]. CBM, a non-catalytic polysaccharide-recognizing module possessed by some GHs, influences enzymes activities by attaching to the substrate [34]. We found that only five GHs containing CBM domains in *P. ananatis* Sd-1. The value is far less than that reported in *C. bescii* DSM 6725 (14) and *C. thermocellum* ATCC 27405 (38). *C. thermocellum* ATCC 27405 is a typical cellulosome production strain [35], which might contain multiple CBM domains for cellulose substrates attachment. Its cellulosome subunits have been identified by the proteomic analysis [8]. *Caldicellulosiruptor bescii* genus are well-studied multifunctional enzymes production strains. Their multifunctional enzymes generally contain one or several CBMs [36]. Correspondingly, a group of glycosidases with CBM3 domains have been identified in the secretome of *C. bescii* when grown on crystalline cellulose. This implied that their enzymes might exist as free enzymes or are incorporated into cellulosomes [37]. In contrast, *P. ananatis* Sd-1 possesses typical cellulase genes encoding endoglucanase, β-glucosidase, and exoglucanase lacking CBM domains. Upon comparison of expression levels, we found that transcripts of all detected genes encoding these enzymes were significantly up-regulated in *P. ananatis* Sd-1 cultured in rice straw-containing medium (at least *P* < 0.05). Higher activities of these enzymes were also all detected in the supernatant of rice straw cultures relative to those in glucose cultures. Correspondingly, we found that endoglucanase (*egl*), exoglucanase (*exg*l), and two β-glucosidase enzymes (*bgl*1 and *bgl*2) all contain signal peptides. These four enzymes as well as another β-glucosidases (Bgl3) could only be identified in supernatant of rice straw cultures. These evidences indicate that the cellulase systems in *P. ananatis* Sd-1 might be composed of free single enzymes, which are soluble and diffuse away from the cell under culture conditions.

Several enzymes, including xylanases and esterases, are needed to completely degrade the hemicellulose polysaccharides [38]. No typical xylanase gene was found, but it was noticed that the CEs number (25) present in *P. ananatis* Sd-1 are strikingly higher than that of all the compared strains, including other eight *P. ananatis* strains. There are nine esterases belonging to CEs in *P. ananatis* Sd-1: Ets1 Y903_RS0120815 and *est*2 Y903_RS0117780 belonging to the CE10 family shared 85 and 75 % similarity with the endo-1,4-beta-xylanase (GenBank: CRH34589.1) and xynB (GenBank: WP_052270754), respectively. Both sequences of the two proteins contain signal peptides. Members of CE10 have been found to possess both carboxylesterase and xylanase activities [39]. In addition, two enzymes from CE10 have been identified in the secretome of *Penicillium purpurogenum* when it was cultured with acetylated xylan substrates [40]. Consistent with these reports, in *P. ananatis* Sd-1, the transcript levels of four detected esterase genes including *est*1 and *est*2 were significantly up-regulated (at least *P* < 0.01) in rice straw cultures, which corresponded with high xylanase-like activity detected in the same cultures. It should be mentioned that the maximum xylanase-like activity reached 25.29 ± 0.87 U/ml in rice straw cultures, which is higher than that was previously reported for cellulolytic fungus [26]. Furthermore, two esterases including *est*2 were only detected in rice straw cultures. These results are consistent with the previous reports, which indicated that *P. ananatis* Sd-1 might possess additional enzymes for efficient breakdown of hemicellulose and further characterization of these enzymes might broaden existing knowledge of hemicellulose degradation system. Interestingly, no CE10 was found in the eight other compared *P. ananatis* strains, except for typical xylanase genes. The difference between *P. ananatis* Sd-1 and other *P. ananatis* strains might be attributed to an evolutionary adaption of *P. ananatis* Sd-1 to the endophytic environment, which was different with that of other compared *P. ananatis* strains [21, 41].

Rice straw saccharification by the *P. ananatis* Sd-1 secretome from rice straw cultures confirmed the existence of its lignocellulosic polysaccharides degradation system. The highest yield of released reducing sugars was attained from the third day’s secretome, whereas the lowest yield was observed from the sixth day’s secretome. These were approximately consistent with results of enzymes activities detection. Analyses indicated that the secretome of *P. ananatis* Sd-1 release higher amounts of reducing sugars than that reported for *Phomaexigua* secretome which used pretreated paddy straw or wheat straw as substrates [42, 43]. These indicated that *P. ananatis* Sd-1 is deserved an in-depth exploration for its enzyme system, such as GHs and CEs.

Although bacterial enzymes associated with lignin degradation are not well characterized in comparison to their fungal counterparts, it has been reported that bacteria have their own unique enzymes for lignin degradation, e.g., Cα-dehydrogenase (*Lig*D), glutathione-dependent β-etherase enzymes (*Lig*E, F, G), demethylase enzyme (*Lig*X), and DyP-type peroxidases [30, 44–46]. Hence it is not surprising that there is no classic fungi lignin degradation enzyme in genome of *P. ananatis* Sd-1. Recent reports of multicopper oxidases demonstrating laccase activities have increased [47, 48]. Secreted catalase/hydroperoxidase, which possess similar heme-containing domains to lignin peroxidases, is associated with strong peroxidase activity and was shown to play an important role in the lignin degradation process in *E. lignolyticus* SCF1 [25]. In *P. ananatis* Sd-1, significantly up-regulated transcript levels of 2 multicopper oxidase genes (*P* < 0.001) and catalase/hydroperoxidase gene (*P* < 0.05) were found to corresponded with higher laccase-like and lignin peroxidase-like activities in rice straw cultures relative to glucose cultures. These results suggest that these enzymes might fulfill similar roles to laccase and lignin peroxidase, respectively, in *P. ananatis* Sd-1. Notably, the maximum value (2.59 ± 0.11 U/ml) for lignin peroxidase-like activity produced by *P. ananatis* Sd-1 in rice straw culture exceeds previously reported values for any fungus or bacterium [49, 50]. Furthermore, the identification of multicopper oxidase (*lac*4) and catalase/hydroperoxidase (*cat*) proteins among secretome of rice straw cultures provides supporting evidence. These data provide a catalog of novel bacterial enzymes participating in the deconstruction of lignin. Indeed, we have cloned one multicopper oxidase gene *lac*4 (Y903_RS0119240) and the recombinant enzyme can efficiently degrade lignin and decolorize dyes in vitro [51].

During fungal degradation of lignin, GMC family oxidoreductases such as glucose oxidase, aryl-alcohol oxidase, methanol oxidase, and pyranose oxidase were reported to participate by providing the hydrogen peroxide required by ligninolytic peroxidases [52, 53]. According to our phylogenetic analysis, the pyranose oxidase enzyme might be the GMC family oxidoreductase equivalent in *P. ananatis* Sd-1. In addition, hydrogen peroxide production is a typical character in the Fenton chemistry pathway. Quinone oxidoreductases were found to drive Fenton chemistry systems participated in lignin degradation in the brown-rot fungus *Gloeophyllum trabeum* [54]. Interestingly, transcript levels of the GMC family oxidoreductase and quinone oxidoreductases in *P. ananatis* Sd-1 were all significantly up-regulated in rice straw cultures relative to glucose cultures (at least *P* < 0.01). These data provide clues that the Fenton chemistry pathway might also exist in bacteria, although this process was shown previously to only exist in fungi. Further research in support of this hypothesis is currently underway.

In our study, it was surprisingly to find that there are nine AAs in *P. ananatis* Sd-1, while they are absent from the other compared strains except for one. Considering their supposed role in lignin degradation [32], the enzyme system in *P. ananatis* Sd-1 might be a new resource for lignin decomposition. Although in this study, we only confirmed two AAs (AA2-Catalase/hydroperoxidase and AA3-GMC family oxidoreductase) were related to lignin degradation, we cannot exclude the possibility that other *P. ananatis* Sd-1 AAs are lignin degradation relevant enzymes. This speculation cannot be verified due to the limited reference information presently available.

Possessing unique enzymes for lignin degradation is an attractive characteristic in a bacterial degradative system. For example, GSTs (*Lig*E, F, G) are able to cleave the β-aryl ether in *Sphingomonas paucimobilis* SYK-6 [44]. According to phylogenetic analysis, four GST genes in *P. ananatis* Sd-1 might possess β-etherase activity. Transcript levels of these four GST genes were significantly up-regulated when rice straw was used as substrate (at least *P* < 0.05). It was not surprising that GST remained undetected in the secretome of *P. ananatis* Sd-1, as none of the four GSTs possesses signal peptides. This is consistent with a report of GSTs in *Sphingomonas paucimobilis* SYK-6 [44]. Our results suggested that a distinct bacteria β-aryl ether degradation pathway might also exist for lignin degradation by *P. ananatis* Sd-1.

Our results confirmed that microbial lignocellulolytic enzymes production is dependent on the carbon source [11]. Growth comparisons of *P. ananatis* Sd-1 in different media indicted poorer growth in rice straw compared to glucose. However, the secreted protein contents from rice straw cultures were higher. These indicated that the discrepancy in enzymes activities was not related to cell density, but rather differences in gene expression in response to the carbon sources.

According to the work in this paper, further studies should focus on the individual enzyme function to aid in development of efficient biological treatment schemes. Elucidation of the mechanistic features of *P. ananatis* Sd-1 lignocellulolytic system is also indispensable for its further biotechnological application.

## Conclusions

This study confirms the strong lignocellulosic biomass degradation capacity of rice endophytic bacterium *P. ananatis* Sd-1. Genomic analysis revealed that *P. ananatis* Sd-1 possesses abundant lignocellulolytic enzymes genes, especially for ligninolytic enzymes relevant genes compared to other cellulolytic and ligninolytic bacteria. A part of these genes transcript levels were found to be highly up-regulated when induced by lignocellulosic substrates, which was corroborated by the secretomic and enzyme activity profiles. In addition, the secretome of *P. ananatis* Sd-1 showed strong saccharification ability toward rice straw. Furthermore, we conclude that the cellulase system of *P. ananatis* Sd-1 might function as free single enzymes. The β-aryl ether degradation and Fenton chemistry pathways might exist in lignin degradation process of *P. ananatis* Sd-1. The discovery of lignocellulolytic system in *P. ananatis* Sd-1 is crucial for a deeper understanding of its degradation mechanisms and the regulation of their extracellular proteins. This work and further evaluation will therefore be a cornerstone for future applications of *P. ananatis* Sd-1 in biofuels production.

## Methods

### Bacterial strains and growth media

The *P. ananatis* Sd-1 (China General Microbiological Culture Collection Centre, no. 6698) is an endophytic bacterium isolated from rice seed in Changsha city of Hunan Province, China [18]. *P. ananatis* Sd-1 colonies were inoculated in Luria–Bertani (LB) broth medium and incubated until the logarithmic growth phase at 30 °C with shaking at 170 rpm. Then 1 ml culture was inoculated into triplicate 100 ml sterile rice straw (pretreated as previously described [18]) or glucose-containing mineral salt media (10.0 g rice straw or glucose, 2.0 g (NH_4_)_2_SO_4_, 1.0 g KH_2_PO_4_, 1.0 g KH_2_PO_4_, 0.2 g MgSO_4_, 0.1 g CaCl_2_, 0.02 g MnSO_4_, and 0.05 g FeSO_4_ in 1000 ml distilled water, pH 7.2) and incubated for 6 days at 30 °C with shaking at 170 rpm.

### Bacterial strain growth measurement

To assess growth of bacteria in rice straw or glucose-containing media, 1 ml samples were periodically withdrawn at 24 h intervals and serially diluted (up to 10^−7^) were proceeded with sterile 0.8 % (*w/v*) normal saline. Thereafter, 1 ml diluted sample was placed on LB medium and spread over the agar surface, and incubated at 30 °C for 24 h. Cell counts were performed on plates containing between 30 and 300 colonies. Results were calculated as log10 CFU/ml. All assays were performed with three replicates.

### Enzyme assays

A total of 2 ml samples were withdrawn from rice straw and glucose cultures periodically at every 24-h interval for lignocellulolytic enzyme activity assays. Endoglucanase and exoglucanase activities were determined according to the method of Ghose using 1 % (*w/v*) sodium carboxymethyl cellulose and Whatman grade 1 filter paper as substrates, respectively [55]. Xylanase-like activity was assayed as described by the method of Bailey et al. using beechhood xylan as substrate [56]. Released reducing sugar from the reaction were determined by the 3,5-dinitrosalicylic acid (DNS) method [57] using glucose or xylose as standard. One unit of enzyme activity was defined as the amount of enzyme that catalyzes the release of 1 µmol reducing sugar as glucose or xylose equivalent per minute under the specified assay conditions. The β-glucosidase activity was assayed as described by Parry et al. [58] using 10 mM *p*-nitrophenyl-β-d-glucopyranoside as substrate. The reaction was terminated by adding 100 µl 1 M Na_2_CO_3_ and the color developed was measured at 405 nm. One unit of enzyme activity was defined as the amount of enzyme required to release 1 µmol *p*-nitrophenol per minute under the conditions of the assay.

Lignin peroxidase-like activity was measured according to the method of Shi et al. by monitoring oxidation of veratryl alcohol to veratraldehyde at 310 nm (ε310 = 9300 mol^−1^ cm^−1^) [59]. Laccase-like activity was measured according to the method of Nakagawa et al. by monitoring oxidation of 2,2,-azino-bis (3-ethylbenzothiazoline-6-sulfonic acid) (ABTS) to ABTS radical at 420 nm (ε420 = 36000 mol^−1^ cm^−1^) [60]. The manganese peroxidase-like activity was measured by monitoring oxidation of 2,6-dimethyl phenol (2,6-DMP) to coerulignone at 469 nm (ε469 = 49,600 mol^−1^ cm^−1^) [59]. One unit of lignin peroxidase-like or laccase-like or manganese peroxidase-like activity was defined as the amount of enzyme needed to produce 1 µmol product per minute. All assays were performed with three replicates.

### Genome sequencing and analysis of *Pantoea ananatis* Sd-1

Genomic DNA of *P. ananatis* Sd-1 was extracted from an overnight culture in LB broth medium using a GeneRay Bacterial Genomic DNA Extraction Kit (GeneRay, Shanghai, China). The genome of *P. ananatis* Sd-1 was sequenced at Novogene Bioinformatics Technology Co., LTD (Beijing, China) using Illumina Hiseq 2000, with a shotgun library of 500 bp insertion size. Prediction and annotation of genes were performed using the Prodigal (Prokaryotic Dynamic Programming Gene-finding Algorithm). Functional annotation was performed by BLASTP algorithm using the public database KEGG, NR, TrEMBL, and SWISS-PROT. The complete genome sequence of *P. ananatis* Sd-1 was deposited at DDBJ/EMBL/GenBank under the accession number AZTE00000000. Genes related to CAZy were performed using the dbCAN CAZyme annotation program from dbCAN pipelines (http://csbl.bmb.uga.edu/dbCAN/index.php) [61] against the Carbohydrate-Active Enzymes database (http://www.cazy.org/) [62]. The output from dbCAN was parsed using the following cutoff values: if alignment length >80aa, using E-value <1e^−5^, otherwise using E-value <1e^−3^. Signal peptides prediction was performed by SignalP v4.0 [63]. The compared CAZy of strains were obtained from Carbohydrate-Active Enzymes database and various references except the 4 partially assembled genomes strains: *P. ananatis* B1-9 [GenBank: CAEJ00000000], *P. ananatis* LMG 2665 [GenBank: JMJJ00000000], *P. ananatis* PA4 [GenBank: JMJK00000000], and *P. ananatis* BD442 [GenBank: JMJL00000000]. After annotation of genes from those four strains genomes, CAZyme annotation of them was performed as previously mentioned.

### Total RNA isolation and cDNA synthesis

Total RNA was extracted from the cells of *P. ananatis* Sd-1 collected from rice straw and glucose-containing medium at the third day using TRIzol reagent (Invitrogen, Carlsbad, CA, USA) according to the manufacturer’s specifications.

Reverse transcription was performed using PrimeScript RT reagent Kit (Perfect Real Time) (Takara, Dalian, China) according to the manufacturer’s instructions.

### Quantitative real-time PCR

The primers (Additional file 7: Table S3) used for qRT-PCR in this study were designed by Beacon Designer 7.7 software. The qRT-PCR reactions were performed on Mx3000P thermal cycler (Stratagene, Santa Clara, CA) and were normalized using 16S rRNA expression levels as internal reference. Reactions were conducted using SYBR^®^ Premix Ex Taq™ II (Tli RnaseH Plus) (Takara, Dalian, China). The applied cycling conditions were as follows: initial denaturation at 95 °C for 30 s, followed by 40 cycles of 95 °C for 5 s, then 60 °C for 30 s, and 72 °C for 30 s. At the end of the amplification of reaction, dissociation curves were performed to verify primer specificity. All reactions were performed in triplicate. Relative transcript levels were determined by the 2^−ΔΔCt^ method [64]: normalizing the gene expression levels in rice straw-containing medium to that in glucose-containing medium, where expression level of the gene in glucose-containing medium was set to one.

### Total secretome protein extraction

Cultures of *P. ananatis* Sd-1 grew in rice straw and glucose-containing media were harvested at every 24-h interval. After filtering, cells and supernatant were separated by centrifugation (4000 rpm, 10 min, 4 °C) and further clarified by filtration through 0.2 µm membranes (Millipore corporation, Billerica, MA). The supernatant protein fraction was concentrated using acetone precipitation [42]. The precipitate protein pellets were dissolved in an appropriate volume of citrate buffer (50 mM, pH 4.8) and used for the saccharification of rice straw. For identification of the secretome protein, the supernatant protein fraction of the third day was precipitated with 10 % (*w/v*) trichloroacetic aid overnight at 4 °C and collected by centrifugation (10,000 rpm, 30 min, 4 °C). The resulting protein pellets were washed three times with 96 % ethanol (*v/v*) and then dried. Protein pellets were resuspended in a sample preparation solution containing 8 M urea, 4 % (*w/v*) 3-[(3-cholamidopropyl) dimethylammonio] propanesulfonate (CHAPS), 40 mM dithiothreitol (DTT). Protein concentrations were determined with the Non-Interference Protein Assay Kit (Sangon Biotech, Shanghai, China).

### Saccharification of rice straw by secretome of *P. ananatis* Sd-1

Saccharification of pretreated rice straw was carried out in screw-capped plastic bottles containing 10 % substrate (*w/v*) and secretome of *P. ananatis* Sd-1 with 0.01 % sodium azide. Saccharification was carried out at 50 °C with shaking at 150 rpm for 72 h. Samples were withdrawn at every 12- intervals for total reducing sugars assay. The yield of released reducing sugar was expressed as mg per gram of dry solid cell mass (mg/gds).

### Secretome protein identification

Twenty-five micro gram of protein samples from each treatment were loaded into a 12 % SDS–polyacrylamide gel for electrophoresis. After destaining, each sample lane was horizontally dissected into 16 fractions and further excised into 1 mm^2^ pieces.

Protein digestion and peptide extraction were performed according to Andreji et al. [65]. Gel fragments were washed once with 50 % (*v/v*) methanol and destained twice for 30 min in acetonitrile 50 % (*v/v*) with 25 mM ammonium bicarbonate. Then incubated with 10 mM DTT in 25 mM ammonium bicarbonate at 56 °C for 1 h and subsequently incubated with 55 mM iodoacetamide in the dark at room temperature for 45 min. Thereafter, gel fragments were digested for 18 h at 37 °C with 12 ng/µl sequencing grade modified trypsin (Promega, Madison, USA) dissolved in 25 mM ammonium bicarbonate and 10 % (*v/v*) acetonitrile solution. Following trypsin digestion, extraction solution containing 66.7 % acetonitrile and 5 % formic acid was added and incubated with sonication for 15 min. Peptides were collected from the extraction solution, dried by vacuum centrifugation, and resuspended in 0.1 % formic acid solution for mass spectrometric analysis.

Samples were analyzed with two technical repetitions each. NanoLC–MS/MS analysis was performed using an EASY-nano LC system (Proxeon Biosystems, Odense, Denmark) coupled online with an LTQ-Orbitrap Velos mass spectrometer (Thermo Scientific, Waltham, MA). Peptides were loaded onto a PepMap C18 trap column (75 μm, 15 cm) (Dionex corporation, Sunnyvale, CA) and eluted using a gradient from 100 % solvent A (0.1 % formic acid) to 35 % solvent B (0.1 % formic acid, 100 % acetonitrile) for 38 min, 35–90 % solvent B for 15 min, and 100 % solvent B for 5 min (a total of 65 min at 200 nL/min). After each run, the column was washed with 90 % solvent B and re-equilibrated with 5 % solvent A. Mass spectra were acquired in positive ion mode applying data-dependent automatic survey MS scan and MS/MS acquisition modes. Each MS scan in the Orbitrap analyzer (mass range = m/z 350–2000, resolution = 60,000) was followed by MS/MS of the seven most intense ions in the LTQ. Fragmentation in the LTQ was performed by high-energy collision-activated dissociation, and selected sequenced ions were dynamically excluded for 30 s. The raw data were viewed in Xcalibur (version 2.1, Thermo Scientific, Waltham, MA), and data processing was performed using Proteome Discoverer (version 1.3 beta, Thermo Scientific, Waltham, MA). The raw files were submitted to a database search using Proteome Discoverer with an in-house sequence algorithm against all predicted CDS of *P. ananatis* Sd-1 described above. The searches were performed with the following parameters: MS accuracy, 10 ppm; MS/MS accuracy, 0.05 Da; two missed cleavage sites allowed; carbamidomethylation of cysteine as a fixed modification; and oxidation of methionine as variable modifications. A minimum of two peptides per protein was accepted for identification. The identification lists from technical repetitions were merged, and repeated protein groups were removed.

## Additional files

10.1186/s13068-016-0439-8 Genome features of *Pantoea ananatis* Sd-1.pdf.
10.1186/s13068-016-0439-8 Carbohydrate-active enzymes identified in the *Pantoea ananatis* Sd-1 genome.xlsx.
10.1186/s13068-016-0439-8 GHs contained multiple CBM domains in genome of *P. ananatis* Sd-1. Blue colors represented linker.png.
10.1186/s13068-016-0439-8 1D-PAGE of proteomes of *P. ananatis* Sd-1 grew on rice straw-containing medium (RS) and glucose-containing medium (G).png.
10.1186/s13068-016-0439-8 Molecular weight and isoelectric focussing of identified proteins. G, proteins identified in the supernatant of glucose-containing medium; RS, proteins identified in the supernatant of rice straw-containing medium. Both the molecular weight and isoelectric point were theoretical obtained from the Proteome Discoverer v.1.3 beta (Thermo Scientific) prediction during the proteins identification.png.
10.1186/s13068-016-0439-8 The identified proteins in proteomes induced by rice straw and glucose substrates.xlsx.
10.1186/s13068-016-0439-8 Primers used for quantitative real-time PCR in this study.xlsx.

## Abbreviations

CAZy: carbohydrate-active enzymes
CDS: protein coding sequences
GHs: glycoside hydrolases
GTs: glycosyltransferases
CEs: carbohydrate esterases
CBMs: carbohydrate bingding modules
AAs: auxillary activities
PLs: polysaccharide lyases
GMC: glucose–methanol–choline
GST: glutathione S-transferase
qRT-PCR: quantitative real-time polymerase chain reaction
nanoLC–MS/MS: nano liquid chromatography–tandem mass spectrometry
pI: isoelectric points
LB: Luria–Bertani
CFU: colony forming units
VA: veratryl alcohol
ABTS: 2,2,-azino-bis(3-ethylbenzothiazoline-6-sulfonic acid)
DNS: 3,5-dinitrosalicylic acid
2,6-DMP: 2,6-dimethyl phenol
DTT: dithiothreitol
CHAPS: 3-[(3-cholamidopropyl) dimethylammonio] propanesulfonate
