# Supplementary material for: Genomic and secretomic insight into lignocellulolytic system of an endophytic bacterium Pantoea ananatis Sd-1
Source: Biotechnol Biofuels. 2016 Feb 2;9:25. doi: 10.1186/s13068-016-0439-8 (PMC4736469; doi:10.1186/s13068-016-0439-8)
Supplement: Supplementary file 1 — 10.1186/s13068-016-0439-8 Genome features of Pantoea ananatis Sd-1.pdf. [file 13068_2016_439_MOESM1_ESM.pdf]

Table S1 Genome features of *Pantoea ananatis* Sd-1

**Features**	**Chromosome**
Genome size (bp)	4,927,500
G+C content (%)	53.34
Total number of genes	4548
Protein coding genes (CDS)	4332
rRNA operons (5S, 16S, 23S)	9
tRNA genes	65
