# Supplementary figures and images for: Genomic and secretomic insight into lignocellulolytic system of an endophytic bacterium Pantoea ananatis Sd-1
Source: Biotechnol Biofuels. 2016 Feb 2;9:25. doi: 10.1186/s13068-016-0439-8 (PMC4736469; doi:10.1186/s13068-016-0439-8)

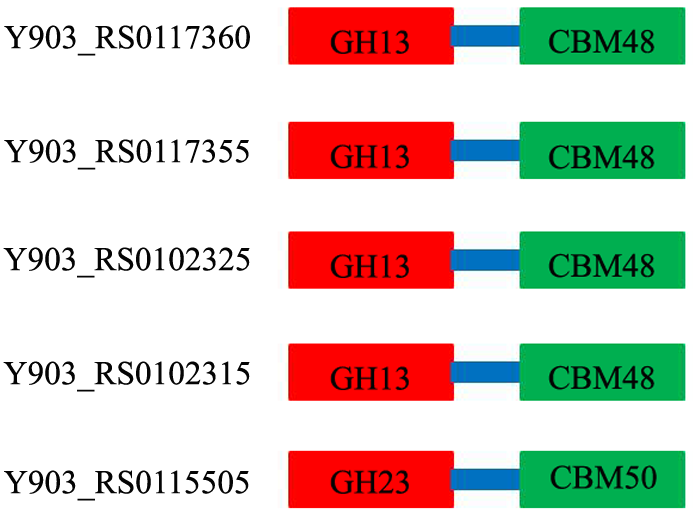

Supplement: Supplementary file 3 — 10.1186/s13068-016-0439-8 GHs contained multiple CBM domains in genome of P. ananatis Sd-1. Blue colors represented linker.png. [file 13068_2016_439_MOESM3_ESM.png]

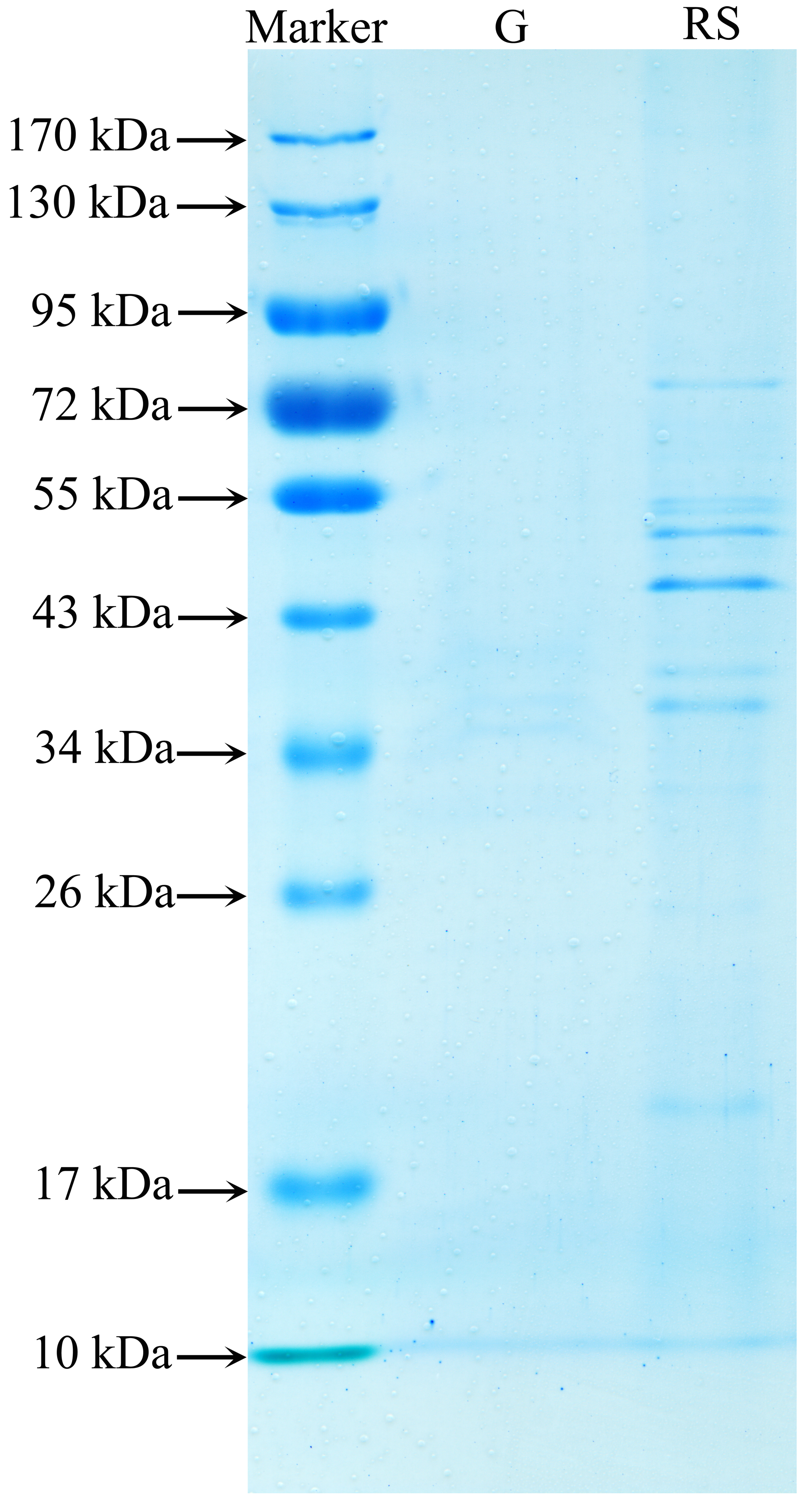

Supplement: Supplementary file 4 — 10.1186/s13068-016-0439-8 1D-PAGE of proteomes of P. ananatis Sd-1 grew on rice straw-containing medium (RS) and glucose-containing medium (G).png. [file 13068_2016_439_MOESM4_ESM.png]
